# On the Association Between Musical Training, Intelligence and Executive Functions in Adulthood

**DOI:** 10.3389/fpsyg.2019.01704

**Published:** 2019-07-30

**Authors:** Antonio Criscuolo, Leonardo Bonetti, Teppo Särkämö, Marina Kliuchko, Elvira Brattico

**Affiliations:** ^1^Department of Cognitive Neuroscience, Faculty of Psychology and Neuroscience, Maastricht University, Maastricht, Netherlands; ^2^Center for Music in the Brain, Department of Clinical Medicine, Aarhus University – The Royal Academy of Music, Aarhus/Aalborg, Aarhus, Denmark; ^3^Cognitive Brain Research Unit, Department of Psychology and Logopedics, Faculty of Medicine, University of Helsinki, Helsinki, Finland

**Keywords:** musical training, cognition, intelligence quotient, working memory, attention, executive functions

## Abstract

Converging evidence has demonstrated that musical training is associated with improved perceptual and cognitive skills, including executive functions and general intelligence, particularly in childhood. In contrast, in adults the relationship between cognitive performance and musicianship is less clear and seems to be modulated by a number of background factors, such as personality and socio-economic status. Aiming to shed new light on this topic, we administered the Wechsler Adult Intelligence Scale III (WAIS-III), the Wechsler Memory Scale III (WMS-III), and the Stroop Test to 101 Finnish healthy adults grouped according to their musical expertise (non-musicians, amateurs, and musicians). After being matched for socio-economic status, personality traits and other demographic variables, adult musicians exhibited higher cognitive performance than non-musicians in all the mentioned measures. Moreover, linear regression models showed significant positive relationships between executive functions (working memory and attention) and the duration of musical practice, even after controlling for intelligence and background variables, such as personality traits. Hence, our study offers further support for the association between cognitive abilities and musical training, even in adulthood.

**HIGHLIGHTS:**

- Musicians show higher general intelligence (FSIQ), verbal intelligence (VIQ), working memory (WMI) and attention skills than non-musicians. Amateurs score in between.

- Significant positive correlations between years of musical playing and cognitive abilities support the hypothesis that long-term musical practice is associated with intelligence and executive functions.

## Introduction

### Musical Training Relies on Executive Functions

Musical training is a multisensory experience engaging multiple cognitive functions and underlying neural networks. Indeed, reading, listening, understanding and performing polyphonic music require the simultaneous processing of sounds and rhythms, higher order perceptual processing and fine sensory-motor coordination (Münte et al., 2002). Long-term musical training engages and trains all those functions on a daily basis and, as a result, musicians seem to improve not only music-related abilities, but also domain-general skills. Hence, musicians show increased auditory perception and production abilities, such as enhanced capacity to detect deviations in complex regularities and tone patterns (Tervaniemi, 2001, 2009; Fujioka et al., 2004; Zuijen et al., 2004; Van Zuijen et al., 2005; Bangert and Schlaug, 2006; Herholz et al., 2009) as well as fine motor control (Krings et al., 2000; Koeneke et al., 2004; Vuust et al., 2005; Kleber et al., 2013; Burunat et al., 2015).

Besides improving listening and sensorimotor abilities closely linked to the musical practice (Schellenberg, 2011), there is also evidence in favor of the *far transfer effect* to non-musical functions. In the literature, *far transfer effects* relates to the influence of musical training on general (not confined to the auditory domain) cognitive functions, such as spatial (Gromko and Poorman, 1998; Rauscher, 2002; Brochard et al., 2004; Sluming et al., 2007), mathematical (Cheek and Smith, 1999), and non-verbal (Forgeard et al., 2008) abilities. Among these, working memory (WM) refers to the ability to retrieve, monitor, analyze, integrate, chunk and recall within a short time span both auditory and non-auditory information (Herholz et al., 2009; Hansen et al., 2013; for reviews, see, e.g., Kraus and Chandrasekaran, 2010; Reybrouck and Brattico, 2015; Schlaug, 2015). In music processing, WM integrates sound events, recollects information from memory systems, links sounds to meaning and to memories, and supports the generation of emotional reactions (Burunat et al., 2014).

Along with cognitive flexibility, response inhibition and interference control, WM is considered one of the fundamental executive functions in humans (Diamond, 2013). Executive functions designate a set of abilities related to updating and manipulating relevant information (WM), inhibiting automatic responses, shifting attention to mental tasks (selective attention), planning, reasoning and decision making (Guare and Dawson, 2004; Perrotin et al., 2008; Garner, 2009; Collins and Koechlin, 2012). Improvements in executive functions and cognitive flexibility by musical training have been observed in Finnish school-age children and these improvements positively correlated to enhanced neural sound discrimination (Saarikivi et al., 2016).

### Effects of Musical Training on General Intelligence

Previous evidence suggests that long-term engagement in musical activities modulates not only executive functions but also general intelligence or *g* (Hansen et al., 2013; see Schellenberg and Weiss, 2013 for a review). In psychological science, *g* has been defined in several ways and assessed using a variety of behavioral tests. Gottfredson (1997, PAG-13) described it as a “very general mental capability that involves the ability to reason, plan, solve problems, think abstractly, comprehend complex ideas, learn quickly and from experience.” *G* seems to rely on similar neural substrates to WM, comprising a network of prefrontal and subcortical regions, along with the anterior cingulate, parietal and premotor regions (Duncan et al., 2000). For similarities at both functional and anatomical levels, Salthouse and Pink (2008) suggested that WM is closely related to *g*. Moreover, the benefits of *far transfer-effects* of musical training extend to the domain of *g* and its quantitative measure, namely intelligence quotient (IQ). For instance, musically trained children show higher IQ as compared to non-trained children (Schellenberg, 2004, 2011).

### Nature or Nurture?

Findings in favor of the link between musical training and cognitive functions led to the notorious debate of nature vs. nurture: i.e., are the observed differences in cognitive abilities only associated with pre-existing neurocognitive differences which predispose people to engage in musical activities (nature), or is the cognitive training promoted by musical activities able to influence cognitive abilities (nurture)? On one side, numerous experimental studies have shown a link between musical training and cognitive development. For instance, in Schellenberg (2004) IQ of a sample of 144 6-year-old children were assessed before and after 1 year of music or drama classes by Wechsler Intelligence Scale for Children–Third Edition (WISC-III). The authors demonstrated that, despite nodifferences in the pre-test for WISC scores, there were greater improvements in WISC scores for children of the music group as compared to the control groups, along with improvements in some of its subscales, such as Verbal Comprehension and Perceptual Organization Indices. Similarly, in Schellenberg, (2011) 106 children aged 9 to 12 (half musically trained and half untrained) were tested with the Wechsler Abbreviate Scale of Intelligence (WASI): trained children showed higher IQ scores than their untrained counterparts. Lastly, beyond IQ assessed with psychological tests, music training in childhood is associated with positive academic achievement (Schellenberg, 2006) and improvements in language-related abilities (Moreno, 2009; François et al., 2013).

Together, these findings highlight an association between musical training and general cognitive abilities in childhood. However, when taking in consideration background variables other than musical training, some authors have shown that pre-existing differences in cognitive abilities, together with differences in children’s and their parents’ personality traits, may contribute in the choice of engaging in musical training and in the duration of such training. In turn, this may ultimately account for differences in cognitive performances in adulthood (Schellenberg, 2006; Corrigall et al., 2013; Corrigall and Schellenberg, 2015). Furthermore, Bonetti and Costa (2017) showed associations between fluid intelligence and music tasks in children aged 4–6 years old with no previous musical training, suggesting a possible innate connection between some musical skills and intelligence that could potentially lead to a higher probability of engaging in musical studies for children with higher IQ. Lastly, by showing that genetic and environmental factors interact in determining music behaviors, such as musical practice and music enjoyment, Butkovic et al. (2015) have further highlighted the need for new investigations to clarify the complex association between music and cognitive development.

### The Current Study

While a consistent corpus of research has focused on child populations, findings in adults are sparse (Brandler and Rammsayer, 2003; Helmbold et al., 2005; Schellenberg and Moreno, 2010). Existing evidence suggests that *far transfer effects* of musical training to general cognitive skills might be related to confounding variables that are usually neglected (Sala and Gobet, 2017), such as personality traits (Corrigall et al., 2013; Corrigall and Schellenberg, 2015).

Aiming to test the hypothesis that long-term musical practice is associated with improved cognitive abilities in adulthood, we assessed intelligence and executive functions of adults with different levels of musical expertise while controlling for background variables such as socio-economic status (SES), age, years of education and personality traits. Differently from previous studies (Brandler and Rammsayer, 2003; Helmbold et al., 2005; Schellenberg and Moreno, 2010; Swaminathan et al., 2017), we adopted the Wechsler Adult Intelligence Scale III (WAIS-III) as intelligence test. WAIS belongs to the family of Wechsler tests, the most used to assess intelligence in the psychological literature (Weiss et al., 2016). To investigate executive functions, we used the Wechsler Memory Scale III (WMS-III) for WM and the Stroop test for selective attention, cognitive flexibility and processing speed. Lastly, personality was assessed by administering participants with the Big Five Inventory questionnaire (BFI) as it was previously done in Corrigall et al. (2013) and in Corrigall and Schellenberg (2015). Our sample includes 101 highly educated Finnish adults (representative of the high education level in Finland; oecd.org) with a mean IQ higher than the average Finnish population [comparing the individual scores with the WAIS norms; (Wechsler, 1997a)].

In line with previous studies (Schellenberg, 2006; Swaminathan et al., 2017, 2018), we expected (i) expert musicians to report higher intelligence and executive functions than non-musicians and (ii) to verify that the positive relationship between the duration of musical practice and cognitive performances would hold even after controlling for potential confounding variables.

## Materials and Methods

### Participants

The participants were part of the broad “Tunteet” research protocol involving a multi-dimensional dataset of brain measures, psychological tests and behavioral data on audition, emotion and musical behfavior. The dataset was obtained from 140 participants recruited among university students or qualified professionals. Further details on this protocol can be found in Burunat et al. (2015, 2016, 2018), Kliuchko et al. (2015, 2016, 2018), Alluri et al. (2017), Bonetti et al. (2017, 2018), and Saari et al. (2018), where some of the participants involved in this study were included. All experimental procedures for this protocol were approved by the Coordinating Ethics Committee of the Hospital District of Helsinki and Uusimaa (approval number: 315/13/03/00/11, obtained on March the 11th, 2012). Furthermore, all procedures were conducted in agreement with the ethical principles of the Declaration of Helsinki.

For the purpose of the current study, we selected only participants who completed psychological and cognitive testing with a trained psychologist (*N* = 114). The other participants did not take part in the testing because their native language was not Finnish, or they did not have enough time to dedicate to the study, and thus other measurements were prioritized. We obtained information on their musical expertise crossing details derived from both a paper and pencil questionnaire (used in previous studies: e.g., Brattico et al., 2009) and an online survey called Helsinki Inventory for Music and Affect Behavior or HIMAB (Gold et al., 2013). Based on those details, subjects were divided into three groups according to levels of musical expertise (or “musicianship” from now on): non-musicians, amateurs, and musicians. Participants were considered musicians when they reported more than 5 years of music practice and considered themselves as musicians. In addition to this, for entering the musicians’ group two criteria had to be matched: a final degree at a music academy or monetarily compensation for their music performance or teaching activities. Participants not matching these parameters, though involved in music activities, were classified as amateurs, and all participants with less than 3 years of musical training entered the group of non-musicians. For the scope of this study, we decided to combine the duration of musical training and the years of musical practice in a comprehensive variable named “years of music playing.” Out of 114 participants, 13 were further excluded because they deviated from normal distribution in one or more background variables (age, years of music playing, years of education, SES). Therefore, only 101 participants were included in this research: 45 were males (44.5%) and 56 were females (55.6%) within the age range of 18–55 years (mean age 28,44 ± 8.26 SD). The SES of participants was assessed by means of the Hollingshead Four-Factor Index (Hollingshead, 1975) included in HIMAB. Details on the participants’ SES, age, gender, years of education, and years of music playing are reported in Table 1, together with personality indices of neuroticism, extraversion, openness to experience, agreeableness, and conscientiousness, as measured by the BFI. Musicians’ and amateurs’ musical background information are provided in Table 2, which includes the starting age of musical training and musical practice, together with the average of weekly hours spent in practicing and in listening to music and years of music playing.

**TABLE 1:** Participants’ background information according to musicianship.

	**Non-musicians**	**mateurs**	**Musicians**	**P-value**	**Beta**	**95% CI LB | UB**	**Partial correlation**
*N of subjects*	51	27	23				
*Gender*	22 M + 26 F	11 M + 16 F	12 M + 14 F				
*Handedness*	5 L	0 L	2 L				
*Age*	29.08 ± 8.91	28.19 ± 8.60	30.17 ± 8.65	0.38	–0.047	−0.022 | 0.012	–0.096
*Years of music playing*	0.55 ± 1.62	6.35 ± 3.28	21.35 ± 7.36	**<0.001**	**0.66**	**0.374 | 0.485**	**0.928**
*Years of education*	17.77 ± 3.51	16.65 ± 3.21	18.95 ± 4.17	0.59	–0.014	−0.029 | 0.024	–0.03
*SES*	35.12 ± 3.19	23.08 ± 4.60	42.89 ± 3.66	0.96	–0.001	−0.007 | 0.007	–0.003
*Neuroticism*	−10.94 ± 11.03	−10.83 ± 10.58	−9.95 ± 10.21	0.45	0.033	−0.01 | 0.015	0.072
*Extraversion*	8.00 ± 9.21	10.48 ± 12.05	9.25 ± 10.41	0.24	0.076	−0.008 | 0.022	0.106
*Openness*	19.19 ± 8.86	19.43 ± 10.24	24.55 ± 6.86	0.59	0.015	−0.014 | 0.017	0.031
*Agreeableness*	15.44 ± 8.54	18.43 ± 7.93	17.10 ± 8.73	0.49	–0.025	−0.018 | 0.012	–0.059
*Conscientiousness*	13.31 ± 9.92	14.43 ± 8.30	10.60 ± 10.05	0.43	0.038	−0.010 | 0.016	00.08

*In order, listed are participants’ gender, handedness, mean and SD for age, duration of musical playing, years of general education, SES (socio-economic status) and personality indices of neuroticism, extraversion, openness to experience, agreeableness and conscientiousness, as measured by the Big Five Inventory questionnaire (BFI). These variables were used as predictors of musicianship in a regression model; associated *p-*value, standardized Beta, 95% confidence interval (CI; lower bound, LB, and upper bound, UB) and partial correlation for each variable are provided. Years of musical playing was the only significant predictor of musicianship and its statistical values are reported in bold.*

**TABLE 2:** Musical background information for amateurs and musicians.

	**Training onset**	**Practice onset**	**Weekly practice hours**	**Listening hours**	**Years of practice**
*Amateurs*	14.04 ± 7.91	11.88 ± 7.21	1.72 ± 3.59	21.85 ± 20.86	**6.35 ± 3.28**
*Musicians*	7.42 ± 4.61	5.36 ± 2.11	15.02 ± 11.12	23.74 ± 15.82	**21.35 ± 7.36**
*P-value*	0.964	0.48	**0.042**	0.311	**<0.001**
*Beta*	0.007	−0.122	**0.285**	0.127	**0.54**
*95% CI LB | UB*	−0.031 | 0.033	−0.056 | 0.027	**0.001 | 0.026**	−0.008 | 0.025	**0.129 | 0.346**
*Partial correlation*	0.008	−0.123	**0.346**	0.176	**0.612**

*In order, means and standard errors of the starting age (in years) of musical training and practice, weekly hours spent in practicing and in listening to music, years of musical practice. *p-*Value, standardized Beta, 95% confidence interval (CI; lower bound, LB, and upper bound, UB) and partial correlation for each variable as reported from a regression model are also provided. Significant statistical values are reported in bold.*

All participants took part in the experiment on a voluntary basis and they were compensated with vouchers to use for culture and sport purposes (e.g., museums, concerts, or swimming pools). All of them were healthy and declared to have no history of neurological or psychiatric disorders. All participants signed an informed consent before the beginning of the experiment and a researcher was present and available for assistance at any time.

### Psychological Tests

#### Wechsler Adult Intelligence Scale III

The WAIS-III is a widely used test for the assessment of adults’ and old adolescents’ intelligence (Wechsler, 1997a). In this study, we used the following eight WAIS-III subtests: Vocabulary, Similarities, Information, Picture Completion, Block Design, Matrix Reasoning, Digit–Symbol Coding, and Symbol Search. The Vocabulary, Similarities, and Information subtests were used to calculate the Verbal Comprehension Index (VCI). The Picture Completion, Block Design, and Matrix Reasoning subtests were used to calculate the Perceptual Organization Index (POI). The Digit–Symbol Coding and Symbol Search subtests were used to calculate the Processing Speed Index (PSI). In addition, these subtests and the Letter-Number Sequencing subtest from WMS-III (which is the same as in WAIS-III) were adopted to estimate the Verbal Intelligence Quotient (VIQ), Performance IQ (PIQ), and full-scale IQ (FSIQ). More details on the tests can be found in Table 3.

**TABLE 3:** Description of the task and the required abilities for the psychological tests administered in the study.

**Test**	**Task**	**Required abilities**
WECHSLER ADULT INTELLIGENCE SCALE III (WAIS-III)		
INFORMATION SUBTEST	Answer general questions	Ability to acquire and retrieve general factual information
SIMILARITIES SUBTEST	Define how two words are similar	Verbal concept formation and reasoning
VOCABULARY SUBTEST	Define the meaning of a word	Word knowledge and verbal concept formation
*COMPREHENSION INDEX*	Combined score of Information, Similarities and Vocabulary	Verbal reasoning
PICTURE COMPLETION SUBTEST	Find what detail is missing in a picture	Perception and recognition of essential visual information
BLOCK DESIGN SUBTEST	Arrange colored blocks to form visual patterns	Ability to analyze and synthesize abstract visual stimuli
MATRIX REASONING SUBTEST	Chooses the missing part that completes the design	Spatial and classification ability, fluid intelligence
*PERCEPTUAL ORGANIZATION INDEX*	Combined score of Picture Completion, Block Design and Matrix Reasoning	Non-verbal/spatial reasoning
DIGIT SYMBOL-CODING SUBTEST	Draw symbols that match numbers paired with the symbols	Visuomotor coordination, psychomotor speed, short-term memory
SYMBOL SEARCH SUBTEST	Match symbols with targets	Visuomotor coordination, psychomotor speed, short-term memory
*PROCESSING SPEED INDEX*	Combined score of Digit Symbol-Coding and Symbol Search	Speed of mental processing
WECHSLER MEMORY SCALE (WMS-III) LETTER-NUMBER SEQUENCING SUBTEST	Recall and mentally rearrange a sequence of digits and letters	Verbal working memory
SPATIAL SPAN SUBTEST	Recall and tap a sequence of spatial positions	Spatial working memory
*WORKING MEMORY INDEX*	Combined score of Letter-Number Sequencing and Spatial span	Working memory
WORD LISTS I SUBTEST	Recall a list of 12 words presented 4 times	Verbal/episodic memory and learning
WORD LISTS II SUBTEST	Delayed recall of the previous word list	Verbal/episodic long-term memory and retrieval
STROOP TEST		
PART 1	Read names of colors written in black ink (RED, BLUE, GREEN…)	Reading
PART 2	Name the color of bars (XXXX, XXXX, XXXX…)	Naming
PART 3	Name the color of words written in different inks (RED, BLUE, GREEN …) – Comparison to Part 1 or 2	Mental flexibility, divided attention, executive functioning

#### Stroop Test

The Stroop effect is measured with the Stroop test and refers to the interference in the reaction time of a task providing conflicting cues. The Stroop effect is used to assess cognitive abilities, such as selective attention, cognitive flexibility and processing speed and, in general, executive functions (Strauss et al., 2006, pp. 477–499). Stroop test scores are calculated based on performances in word reading and color naming tasks. The word reading and color naming are control tasks where the subject is asked to just (i) read the color words printed in black ink and (ii) name the colors of given bars printed in different inks. In the third task, the subject is shown the color words printed in different ink (conflicting the semantic meaning of the word) and is asked to name the colors in which the words are printed. Since word reading is an automatic process, performance in this task requires the subject to inhibit the reading while focusing on the color naming.

Typically, the Stroop effect is derived by comparing the correct responses and performance times of the third task to either one of the control tasks by subtracting the control task from the third task. In effect, this subtraction leaves the actual cognitive process we are interested in (the “cost” of inhibiting the response to the automatic word reading process in the third task). Thereby, the Stroop variable used for this study corresponds to the subtraction of the reaction time obtained in the interference task minus the reaction time obtained in the color naming task. The higher the value, the higher the effort needed to selectively filter out unattended information and focus on attended ones.

#### Wechsler Memory Scale III

The WMS-III is a neuropsychological test designed to measure different memory functions (Wechsler, 1997b). In the present study, we administered the following four WMS-III subtests: Letter-Number Sequencing, Spatial Span (forward and backward), Wordlists I, and Wordlists II, which measure, verbal, spatial, and episodic memory components, respectively. Letter-Number Sequencing and Spatial Span were used to calculate the Working Memory Index (WMI). More details on the tests can be found in Table 3.

#### Big Five Inventory

The BFI contains 44 items designed to measure an individual on five main dimensions of personality: Openness to experience, Conscientiousness, Extraversion, Agreeableness, and Neuroticism (John and Srivastava, 1999). Items are rated on a five-point scale (where 1 corresponds to strongly disagree and 5 to strongly agree), and the score for each personality dimension corresponds to the average rating for the relevant items. Examples of the multiple possible choices for the item: “*I see myself as someone who*…*”* are: *“Is talkative,” “Is reserved,” “Is full of energy,” and “Can be tense.”*

### Procedure and Statistical Analysis

The participants were invited to the Advanced Magnetic Imaging (AMI) laboratory for the neuroimaging session of the broad Tunteet project (coordinated by EB). There, before and after the brain scanning session, they were administered the following tests by a graduate student of psychology under supervision of a licensed and expert psychologist (TS): Stroop test, WMS-III, and WAIS-III. In another session taking place at the Biomag laboratory at Helsinki Central University Hospital, the same participants were invited for the second part of the brain scanning and personality data were collected by administering the complete BFI. The total duration of each experimental session was around 3 h. The psychological tests, in total, did not last longer than 2 h. For the purposes of the present study we only used the results of the psychological tests.

Before testing for group differences in cognitive abilities along musicianship, we controlled that there were no significant group differences in background variables. Therefore, two regression models were performed: the first includes background variables of age, years of general education, SES, personality traits variables and years of music playing as predictors of musicianship (classification in non-musicians, amateurs, and musicians); the second model was performed for amateurs and musicians only and included music-background variables such as onset of musical training and musical practice, average of weekly hours spent in practicing and on listening to music, together with years of music playing as predictors of musicianship. By doing so, we obtained the relative contribution of each variable in predicting group differences when holding constant the others.

Age, years of general education, SES and personality traits variables were normally distributed across participants. Because years of music playing was not normally distributed, we decided to square-root the variable and use its transformation in the analyses. Results and means values for each variable of the first regression model are provided in Table 1, whereas the others are provided in Table 2. The means displayed for years of music playing are the original values (not the square-root transformed).

In order to test for group differences along cognitive abilities, we performed Multivariate analysis of variance (MANOVA) inserting musicianship as between-subjects factor and the main indices of the cognitive tests scores (Stroop, WMS-WMI, and WAIS-FSIQ) as dependent variables. Having more than two dependent variables and because they significantly correlated with each other (FSIQ-WMI: *r* = 0.628, *p* < 0.001; FSIQ-Stroop: *r* = −0.337, *p* = 0.001; WMI-Stroop: *r* = −0.327, *p* = 0.001), we opted for MANOVA. Indeed, such statistical test is able to take the relationship between dependent variables into account (Warne, 2014). Assumptions of linearity and absence of collinearity were tested before proceeding with the analysis. A separate ANOVA was then performed to examine the differences among groups along subtests of the WAIS-FSIQ, namely WAIS-VIQ and WAIS-PIQ. *Post hoc* tests with Bonferroni correction were performed for both the MANOVA and ANOVA models to avoid false positive discoveries while calculating group comparisons. The Bonferroni-adjusted alpha level for *post hoc* tests was obtained by dividing the standard alpha at 0.05 by the number of comparisons [defined as *N*(*N*−1)/2]. In our case, with 3 groups and 3 variables, there were 9 comparisons; thus, the alpha level was reduced at 0.0056. To be noted, the *p-*values reported in the Results section are Bonferroni-corrected *p-*values, so that a corrected *p* < 0.05 corresponds to a non-corrected *p* < 0.005 and hence is interpreted as a significant effect.

To deepen the exploration of the relationship between musical practice and cognitive abilities, and to control for the influence of potential confounding variables, we performed three backward stepwise linear regression analyses inserting background variables of age, years of education, years of music playing and the five personality trait indices as predictors of FSIQ, WMI, and Stroop, respectively. By doing so, we would obtain the unique contribution of each of the variables, and of music practice, in predicting the variance observed in the cognitive test scores. Backward stepwise regression starts with a saturated model and gradually eliminates (stepwise) variables from the regression model in order to find the predictors that best explain variance in the dependent variable. Therefore, multiple models are generated until model fit cannot be further improved.

Because of missing data along some of the demographic variables, these models only included 60 participants from our sample (20 subjects per group). Therefore, to estimate curve fit along our whole sample we performed three further independent linear regression models by inserting years of music playing as the only predictor of, respectively, FSIQ, WMI, and Stroop. Lastly, to estimate the partial correlation of each of the cognitive measures to musical practice, FSIQ, WMI, and Stroop were included in the same model and regressed against years of music playing. Models’ effect sizes are always reported as adjusted *R*^2^.

## Results

### Musicians, Amateurs and Non-musicians

As compared to amateurs, musicians had spent more years and hours practicing an instrument. Musical background information for amateurs and musicians is provided in Table 2, along with mean, SD and the associated *p, B*, partial correlation values and 95% confidence interval (CI) for the group comparisons.

**FIGURE 1:**
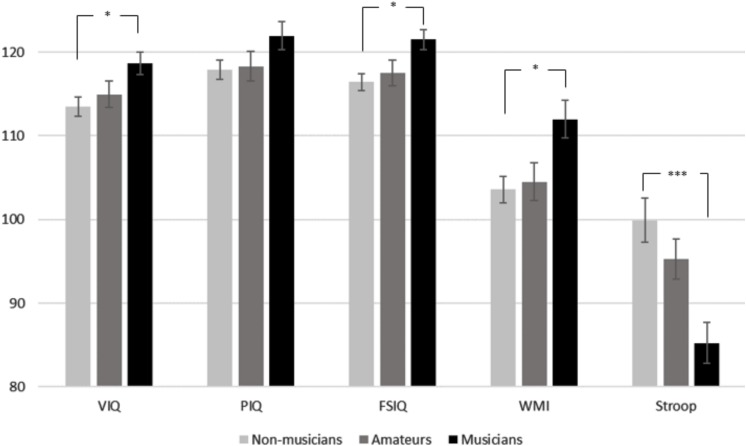
Cognitive scores means along Wechsler Adult Intelligence Scale III (WAIS-III) subscales (in order: Verbal IQ, Performance IQ, Full Scale IQ), Working Memory Index from WMS and Stroop test reported according to musicianship. Bars show confidence intervals (CI). Musicians performed better in all tests compared to other groups. To be noted, Stroop values refer to reaction times: smaller reaction times indicate better performance. Alpha level is Bonferroni-corrected, so that *p* < 0.05 is a significant effect; ^*^p ≤ 0.05, ^∗∗^*p* ≤ 0.01, and ^∗∗∗^*p* ≤ 0.001.

Despite the absence of differences in background variables, musicians performed better in all cognitive tests as compared to the other groups, as shown in the histogram in Figure 1 and in Table 4. The MANOVA performed to compare participants’ FSIQ, WMI, and Stroop cognitive scores exhibited a significant group effect: Pillai’s Trace [*F*(2,98) = 2.889, *p* = 0.01]. The test of between-subject effects reported significant group differences in WAIS-FSIQ [*F*(2,98) = 4.00, *p* = 0.021, adjusted *R*^2^ = 0.057], WMS-WMI [*F*(2,98) = 4.11, *p* = 0.019, adjusted *R*^2^ = 0.059), Stroop [*F*(2,98) = 6.68, *p* = 0.002, adjusted *R*^2^ = 0.102] as provided in Table 4 (on the left side). The effect sizes of these adjusted *R*^2^ are moderate (Cohen, 1988). A separate ANOVA model was performed to assess group differences along VIQ and PIQ and reported significant differences for the former only: VIQ [*F*(2,98) = 3.46, *p* = 0.035]; PIQ [*F*(2,98) = 1.95, *p* = 0.148]. Results are provided on the right side of Table 4.

**TABLE 4:** Mean and statistical group comparisons along cognitive abilities.

**Means of cognitive abilities per group**					
	**WAIS-FSIQ**	**WMS-WMI**	**Stroop**	**WAIS-VIQ**	**WAIS-PIQ**
*Non-musicians(NM)*	116.45 ± 1.01	103.57 ± 1.61	99.91 ± 2.65	113.51 ± 1.17	117.9 ± 1.16
*95% CI*	±0.28	±0.44	±0.72	±0.32	±0.32
*Amateurs(A)*	117.52 ± 1.50	104.52 ± 2.24	95.29 ± 2.43	114.96 ± 1.56	118.33 ± 1.78
*95% CI*	±0.57	±0.85	±0.92	±0.59	±0.67
*Musicians(M)*	121.5 ± 1.22	112 ± 2.29	85.22 ± 2.45	118.67 ± 1.38	121.96 ± 1.69
*95% CI*	±0.50	±0.96	±1.00	±0.56	±0.69

***Mean differences of cognitive abilities***					

*A – NM*	1.07	0.95	−4.62	1.45	0.431
*M – A*	4.00	7.13	10.06	3.70	3.62
*M - NM*	5.07	8.08	−14.69	5.16	4.06

	***Between-subject MANOVA results***			***Between-subject ANOVA results***	
*F(2,98)*	4.00	4.11	6.68	3.46	1.95
*p*	**0.021**	**0.019**	**0.002**	**0.035**	0.148
η^2^*p*	0.057	0.059	0.102	0.046	0.018
ω*p*^2^	0.56	0.058	0.101	0.046	0.018

	***Post hoc p (Bonferroni corrected)***			***Post hoc p (Bonferroni corrected)***	
*A – NM (p)*	1.000	1.000	0.683	1.000	1.000
*95% CI*	−3.10 | 5.23	−5.71 | 7.61	−13.9 | 4.65	−3.14 | 6.04	−4.51 | 5.37
*M – A (p)*	0.157	0.093	0.087	0.297	0.398
*95% CI*	−0.96 | 8.97	−0.8 | 15.07	−21.12 | 0.99	−1.71 | 9.11	−2.20 | 9.45
*M – NM (p)*	**0.018**	**0.018**	**0.001**	**0.030**	0.172
*95% CI*	**0.68 | 9.46**	**1.06 | 15.11**	−**24.47 | −4.90**	0.380 | 9.93	−1.08 | 9.20

*Top: means, standard errors and mean difference for the scores of Verbal Intelligence Quotient (VIQ), Performance Intelligence Quotient (PIQ), Full-Scale Intelligence Quotient (FSIQ), Working Memory Index (WMI), and Stroop for participants grouped into non-musicians, amateurs and musicians. To be noted, Stroop values refer to reaction times: smaller reaction times indicate better performance. Bottom: on the left side, results of the Multivariate analysis of variance (MANOVA) performed to compare musicians (M), amateurs (A) and non-musicians (NM) reporting significant differences along Full Scale IQ (WAIS-FSIQ), Working Memory Index (WMS-WMI), and Stroop test. On the right side, results of a further ANOVA showing the differences between VIQ and PIQ scores. *Post hoc* comparisons and *p-*values corrected for Bonferroni correction are provided at the end of the table for both models. Significant statistical values are reported in bold.*

*Post hoc* tests controlled by Bonferroni correction reported significantly higher values in favor of musicians as opposed to non-musicians for all the different tests: FSIQ (*p* = 0.018), WMI (*p* = 0.018), Stroop (*p* = 0.001), and VIQ (*p* = 0.030) as provided in Table 4. In turn, amateurs did not differ significantly from musicians and non-musicians in either of tests.

To deepen our understanding of the relationship between musical training and cognitive abilities, we performed stepwise backward linear regression modeling by inserting 8 demographic variables (age, years of music playing, years of general education and personality indices of neuroticism, extraversion, openness to experience, agreeableness and conscientiousness) as predictors of FSIQ, WMI, and Stroop (independently). Backward regressions generated in all cases 7 models in which all variables mentioned above were excluded one-by-one except for years of musical practice, which resulted, in all the cases, the only significant factor associated with FSIQ [*F*(1,59) = 6.76, *p* = 0.012, partial-correlation = 0.321, β = 0.321], WMI [*F*(1,59) = 7.23, *p* = 0.009, partial-correlation = 0.330, β = 0.330], and Stroop [*F*(1,59) = 6.81, *p* = 0.012, partial-correlation = −0.324, β = −0.324].

This approach evidenced that (i) when holding constant the other background variables, years of music playing was the only factor significantly associated with the three cognitive measures (FSIQ, WMI, and Stroop). Besides, by excluding the other factors from the model, we found that (ii) years of music playing was the only predictor necessary to significantly explain the variance in the dependent variables.

**FIGURE 2:**
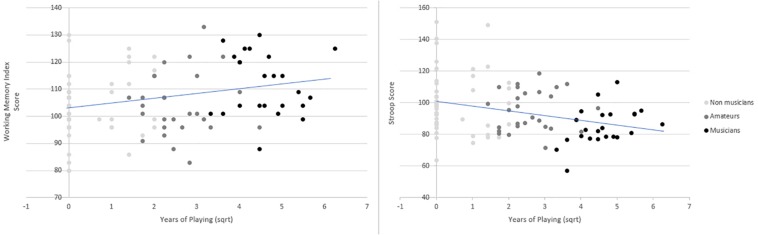
Curve fit showing, on the left, the positive relationship between the variable Years of Music Playing (as square root transformed variable) and Working Memory Index score from Wechsler Adult Intelligence Scale III (WAIS-III) test. On the right, the negative relationship between Years of Playing and Stroop Index score. *Y*-axis corresponds to the individual WMI and Stroop scores, respectively.

Because of missing values in some background variables, not all of the participants were included in these regression models. When regressed independently against years of music playing, WMI and Stroop showed a significant association: WMI [*F*(1,99) = 7.80, *p* = 0.006, adjusted *R*^2^ = 0.064, β = 0.27], Stroop [*F*(1,99) = 8.46, *p* = 0.004, adjusted *R*^2^ = 0.069, β = −0.28], and FSIQ [*F*(1,99) = 3.37, *p* = 0.069, adjusted *R*^2^ = 0.023, β = 0.18]. Figure 2 represents the curve fit for years of music playing with WMI and Stroop: WMI showed a positive association with music playing duration, whereas Stroop showed a negative relation.

To test for the unique association of each of the cognitive variables with years of music playing, an additional model included the three cognitive variables (FSIQ, WMI, and Stroop) and regressed them against years of music playing. This model resulted significant [*F*(3,97) = 4.55, *p* = 0.005, adjusted *R*^2^ = 0.096]. When holding constant the other cognitive variables, WMI and Stroop were still significantly associated with years of musical playing, whereas FSIQ was not: WMI (*p* = 0.041, β = 0.24), Stroop (*p* = 0.022, β = −0.24), and FSIQ (*p* = 0.70, β = −0.05). Standardized coefficients, significance levels, 95% CI and partial correlation values of the regression model for the three cognitive variables (FSIQ, WMI, and Stroop) are provided in Table 5. Furthermore, we repeated the regression excluding the zero values for the variable Years of Music Playing. This operation resulted in exclusion of 36 non-musicians scoring less than 1 in the square-root transformed variable years of music playing, hence in a consistent decrease of statistical power. In spite of this, we could still observe tendencies for an association between cognitive measures of FSIQ, WMI, and Stroop and Years of playing: WMI [*F*(1,64) = 2.90, *p* = 0.094, adjusted *R*^2^ = 0.028, β = 0.208], Stroop [*F*(1,64) = 4.40, *p* = 0.04, adjusted *R*^2^ = 0.05, β = −0.25], and FSIQ [*F*(1,64) = 3.55, *p* = 0.064, adjusted *R*^2^ = 0.038, β = 0.23].

**TABLE 5:** Regression model for cognitive abilities. Standardized coefficients for the regression model where Stroop, FSIQ and WMI were included and regressed against the variable “years of music playing.”

**Standardized coefficients**					
	**Beta**	***t***	**Sig.**	**95% CI**	**Partial correlation**
*(Constant)*		0.537	0.593	−5.221 | 9.09	
*Stroop*	–0.241	–2.328	**0.022**	−**0.049 |** −**0.004**	−**0.230**
*FSIQ*	–0.047	–0.386	0.700	−0.072 | 0.270	−0.039
*WMI*	0.238	2.067	**0.041**	**0.001 | 0.072**	**0.205**

*Beta and *t-*values, as well as significance levels and 95% confidence intervals (CI) are provided for each of the variables when holding constant the others. Significant statistical values are reported in bold.*

## Discussion

The aim of the present study was to investigate the relationship between musical training and higher-order cognitive functions. Although several studies have highlighted anatomical and functional differences between the brains of expert musicians and non-musicians, only few studies have investigated intelligence and executive functions in adults with long-term musical training controlling for possible confounding variables. Our results contribute to the literature by showing that adults exposed to professional long-term musical training outperform adult non-musicians in standardized cognitive tasks designed to measure general intelligence (*g*), WM and attentive abilities and that these group differences are not associated with any of the examined background variables except for duration of musical playing.

Specifically, when testing group differences with analysis of variance we found significantly higher performance in musicians compared to non-musicians in the cognitive tests’ general indices of WMI, FSIQ, and Stroop and significantly greater scores. Slightly less strong effects were obtained in one subscale of the WAIS-III, assessing (VIQ). Moreover, by using regression models we noticed an association between all participants’ cognitive abilities and years of music playing, which, however, did not explain all of the observed variance. To be noted, this association might not necessarily be linear because when removing the participants completely lacking any musical background, the significance threshold of the association was not reached. Nevertheless, overall these findings converge to demonstrate a positive relationship between musical training and cognitive functions. As proposed by Schellenberg (2006), this relationship could be seen as a continuum dependent on the duration and intensity of training. These findings are in line with previous research (both correlational and experimental) showing associations between musical training and intelligence measures (Chan et al., 1998; Gromko and Poorman, 1998; Cheek and Smith, 1999; Hetland, 2000; Brandler and Rammsayer, 2003; Brochard et al., 2004; Schellenberg, 2004, 2006; Swaminathan et al., 2017, 2018). For instance, a previous study with children conducted by Schellenberg (2006) reported an association between musical training and cognitive abilities (VIQ, FSIQ). Lastly, the increased VIQ we found when comparing musicians to non-musicians might be in line with previous findings, which associated music training with improvements in verbal abilities, such as reading (Anvari et al., 2002; Kraus et al., 2014) and phonology (Francois et al., 2015; see Moreno, 2009 for a review). However, differently from previous studies, we show that these effects are not affected by potential confounding variables: indeed, by controlling for age, education years, SES and personality variables, we demonstrate that the relationship between executive functions and years of music playing is statistically significant. In particular, although not explaining all of the variance, we found a positive effect for WM: the longer the musical practice, the higher the WM functions. In turn, Stroop attentive scores show a negative slope: the longer the musical practice, the shorter interference time, the bigger the attentive abilities.

Together with previous studies, our results allow us to argue that cognitive benefits associated with music practice might be evident along the lifespan. In accordance with previous evidence, we argue that ‘widespread effects of musical training on cognitive processing might occur because music lessons train attentional and executive functioning, which benefit almost all cognitive tasks’ (Hannon and Trainor, 2007). It is important to consider, though, that additional variables not considered in the present study, such as genetic factors and parental personality traits, might have had a relevant influence on the choice of our participants to engage in and persist with musical training (Mosing et al., 2014; Mosing and Ullen, 2018), as well as in the development of their cognitive abilities, as pointed out by previous studies (Butkovic et al., 2015; Corrigall and Schellenberg, 2015). Moreover, cognitive advantages might be evident particularly for those individuals who take music lessons and/or play music in addition to other academic and studying activities. Indeed, in a previous study the cognitive effects of musical training were not visible in participants who studied only music (Schellenberg, 2011). In contrast, all participants in our sample were recruited among university students and qualified professionals and reported a mean IQ higher than the average Finnish population. This long academic background might be the key difference between the present and previous studies. Because musicians differed from the other participants only regarding their musical expertise and given the positive relation between executive functions (WM, Stroop-derived attentive score) and length of musical training, our results suggest that cognitive abilities might be influenced by musical practice.

We suggest that the observed differences in cognitive performance might represent the behavioral manifestations of brain differences identified when comparing musicians with non-musicians. Indeed, neuroimaging and neurophysiological studies showed stronger and faster neural responses (especially to sounds) and enlarged neuroanatomical structures in musicians as compared with non-musicians, particularly in (pre)motor, auditory, prefrontal and visual regions (Münte et al., 2002; Gaser and Schlaug, 2003; Pantev et al., 2003; Zatorre and McGill, 2005; Hyde et al., 2009; Zuk et al., 2014; Baer et al., 2015; Bonetti et al., 2017, 2018). These modifications of brain functionality and anatomy have been associated with use-dependent regional growth of neuronal cells engaged throughout the training and their structural adaptation in response to the intense environmental demands of music practice (for reviews, see Kolb and Muhammad, 2014; Reybrouck and Brattico, 2015).

To conclude, our study highlights an association between musical training and cognitive abilities. We showed that adult participants with similar educational background but varying in their musical expertise exhibited differences in intelligence, working-memory and attentive abilities and that executive functions are significantly associated with the duration of music practice.

## Ethics Statement

Coordinating Ethics Committee of the Hospital District of Helsinki and Uusimaa (approval number: 315/13/03/00/11, obtained on March the 11th, 2012).

## Author Contributions

EB conceived the study, prepared the ethics permission, coordinated the data collection, designed the initial statistical analyses, and fully edited the final version of the manuscript. AC conducted the final statistical analyses together with LB and wrote the initial version of the manuscript. MK contributed to the data collection, experimental design as well as coordinated the data scoring, and ensured the data quality. TS selected the psychological tests, and supervised the data collection and scoring. All authors contributed to writing and approved the final version of the manuscript.

## Conflict of Interest Statement

The authors declare that the research was conducted in the absence of any commercial or financial relationships that could be construed as a potential conflict of interest.
